# A 6-Week Nine-Square Exercise Programme for Collegiate Athletes with Chronic Ankle Instability: A Randomised Controlled Trial

**DOI:** 10.21315/mjms2022.29.6.10

**Published:** 2022-12-22

**Authors:** Kanok-On Thanasootr, Torkamol Hunsawong, Uraiwan Chatchawan, Wantana Siritaratiwat

**Affiliations:** 1 School of Physical Therapy, Faculty of Associated Medical Sciences, Khon Kaen University, Thailand; 2 The Research Centre in Back, Neck, Other Joint Pain and Human Performance (BNOJPH), Khon Kaen University, Thailand

**Keywords:** ankle injuries, postural control, rehabilitation, sport medicine, training programme

## Abstract

**Background:**

Individuals with chronic ankle instability (CAI) have poor postural stability, functional limitations and low quality of life. Although nine-square exercise can improve postural control, there is limited evidence demonstrating whether it can function as an alternative CAI rehabilitation programme. This study aimed to determine the effects of nine-square exercise on postural stability and self-reported outcomes in individuals with CAI.

**Methods:**

Eighteen male collegiate athletes with CAI participated in either a 6-week nine-square exercise or a control group (*n* = 9 per group). At baseline and post-intervention, the participants undertook clinical tests to measures dynamic and static postural control, and self-reported outcomes regarding ankle stability and function.

**Results:**

Within-group differences, the nine-square exercise group experienced improved dynamic postural control (*P* = 0.004), static postural control (*P* = 0.001) and self-reported outcomes (*P* < 0.05). For the control group, only static postural control improved (*P* = 0.018). Post-intervention, the nine-square exercise group experienced significant improvements in dynamic postural control (*P* < 0.001), ankle stability (*P* = 0.002) and functional ability (*P* < 0.05) relative to the control group.

**Conclusion:**

These findings suggest that the nine-square exercise can offer an alternative rehabilitation programme for improving postural control, self-perceived ankle stability and functional ability in CAI.

## Introduction

A lateral ankle sprain (LAS) is a common ankle injury with high rates of recurrence; it can develop into chronic ankle instability (CAI) in physically active individuals and young athletes who participate in either team sports (such as basketball, football, volleyball and rugby) or individual sports (such as badminton and running) (1, 2). CAI refers to chronic residual symptoms including recurrent LAS and feelings of ankle joint instability or giving way (1). Nine percent to 25% of high school and collegiate athletes with a previous index LAS are expected to develop CAI (1–2). The multifaceted pathology of CAI leads to impairments such as limited range of motion, impaired static and dynamic postural stability, impaired sensorimotor function, insufficient muscle strength, impaired self-perceived functional ability (3) and change in gait initiation patterns similar to those of elderly individuals and indicative of Parkinson’s disease (4). These impairments contribute to post-traumatic ankle osteoarthritis and negatively impact health-related quality of life (1, 3).

Previous studies on interventions for CAI have observed that a combination of balance and strength training can improve postural control, strength and self-reported outcomes in patients with CAI (5–7). Balance training mainly uses perturbations that affect static postural stability and/or the centre of gravity (COG) while an individual maintains a single-leg stance on an unstable surface (7–8). Notably, previous studies also reported issues with dynamic postural stability related to functional ability in individuals with CAI during gait initiation, walking or running (7, 9). These findings highlight the value of functional-based approaches. For example, gait training on a treadmill improved both gait pattern and self-reported function (7, 9). However, because such approaches require expensive equipment, it may be impossible to implement them outside hospitals or research centres.

Nine-square exercise is a simple and feasible gait training method that can potentially improve postural control and functional ability in individuals with CAI. Nine-square exercise or nine-matrices exercise, is a functional-based training method established by Krabuanrat to improve postural stability, reaction time, cardiopulmonary function, endurance and agility (10). In this method, an individual steps in multiple directions, walks forward-backward and turns around while shifting from a wide to a narrow base of support. During this process, they also increase their walking speed from slow to fast. The only equipment needed is a square mat, which is inexpensive, versatile and convenient (10). Previous studies demonstrated that nine-square exercise improves postural stability, muscle strength and mental health among older adults and increased agility and coordination in young athletes (10–12). Nine-square exercise may simulate the sensorimotor system and lower response latency to restore balance strategies, particularly the counterbalance of perturbations (10–12).

However, there is no evidence that nine-square exercise can significantly benefit individuals with CAI. Therefore, this study aims to primarily determine whether nine-square exercise improves dynamic postural control in these individuals. The secondary aim is to determine the effects of nine-square exercise on static postural control and self-reported ankle stability and functionality.

## Methods

### Study Design

We conducted a two-arm, single-blinded randomised controlled trial to evaluate the effects of a 6-week nine-square exercise programme on postural control and self-reported outcomes in individuals with CAI.

### Participants

We recruited 18 male collegiate athletes who participated in endurance or resistance training for at least 1.5 h per week (8, 13). Participants were recruited through sports clubs at a local university. Following the guidelines of the International Ankle Consortium, our inclusion criteria were as follows: All participants had experienced at least one LAS, the initial LAS had occurred more than 12 months before our study began, and at least two episodes of giving way had occurred within the previous 6 months (14). All participants scored ≤ 25 on the Cumberland Ankle Instability Tool (CAIT) (15) and ≤ 85% on the Foot and Ankle Ability Measure sports (FAAM-Sports) subscale (16) and all had a positive single-leg balance test (17). For participants with bilateral CAI, the limb with the lower CAIT score was used for the data analysis (8, 13). We excluded participants who met any of the following criteria: an acute injury of the lower extremities within the previous three months; previous surgery or fracture in the lower extremities and any other conditions that could affect balance control, such as visual, vestibular or neurological pathologies (14). The University Ethics Committee in Human Research approved the study protocol. Prior to data collection, all participants provided written informed consent.

### Sample Size

We calculated the sample size using a formula for the analysis of covariance in randomised clinical trials (18). Following Cruz-Diaz et al. (8), we selected the power of 80%, the alpha level of 0.05 and the mean difference of the modified Star Excursion Balance Test in the posteromedial direction (mSEBT-PM) of 3.13%. The estimated sample size was seven participants per group. We also predicted a 10% drop-out rate. Therefore, our optimal sample size was nine participants per group.

### Procedure

**Figure 1:**
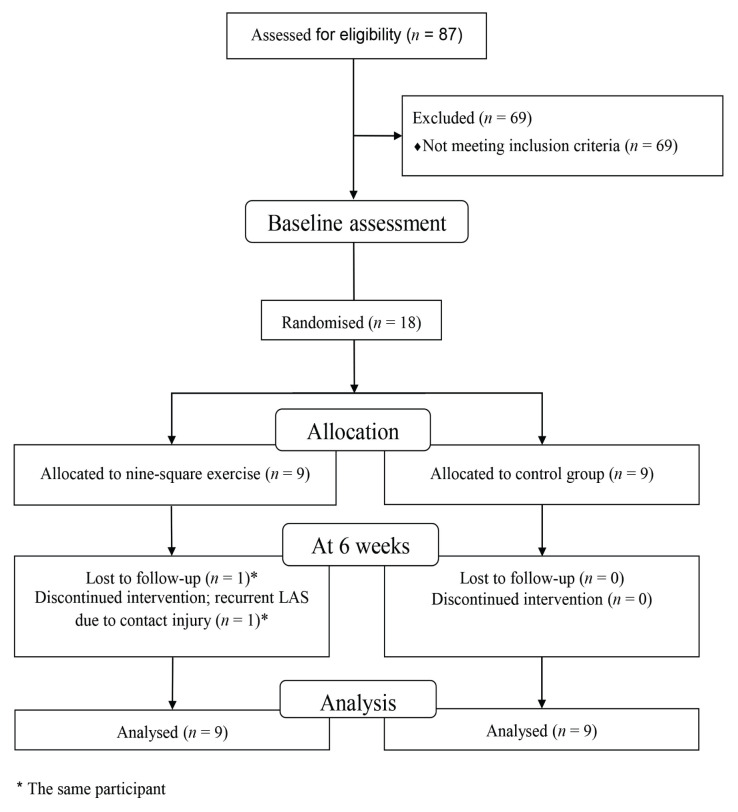
The study’s flow chart

Prior to data collection, we estimated the intra-rater reliability of the selected clinical balance tests and obtained good reliability for the mSEBT-PM (intraclass correlation coefficient or ICC [3, 3] = 0.84) and excellent reliability for the foot lift test (FLT) (ICC [3, 3] = 0.99). We collected baseline and post-intervention (at 6 weeks) scores for all outcomes. A physiotherapist (TH) with 10 years of experience in management of ankle and foot disorders in athletes conducted the tests on all participants. This primary assessor was blinded to group allocation and not involved in the rehabilitation. After the baseline assessment, a research assistant (who was not an author) randomly assigned participants to either the nine-square exercise group or the control group (Figure 1) using sealed opaque envelopes from a randomised computer-generated stratified block (available at: https://www.sealedenvelope.com/simple-randomiser/v1/lists) with types of sports (team sports and individual sports) and age ranges (18 years old–29 years old and 30 years old–40 years old). All participants continued their usual activities after group allocation and we gave each of them a self-care guidebook for CAI.

### Interventions

During the 6-week study protocol, an experienced physiotherapist (KT) with intensive training in nine-square exercise taught and supervised all exercise sessions.

#### Nine-Square Exercise Group

Nine-square exercise is a multi-directional stepping method that requires postural stability, coordination, endurance and functional mobility, and it is carried out on a 90 cm × 90 cm mat (10). We controlled the walking speed of each participant with a metronome of 80 beats–120 beats per min (19). The participant began by taking a step on the non-dominant limb and then completed three repetitions of each pattern (4, 10). We determined a progressive level for nine-square exercise by analysing a participant’s pain intensity, performance during exercise and rate of perceived exertion (RPE), ranging from 11 (fairly light) to 14 (somewhat hard) (20). The sequential pattern of nine-square exercise is as follows: forward-backward step, lateral side step, ‘X’ step, diamond step, double inverted-triangle step, triangle step, ‘V’ step, star radius step and diagonally crossing step (10).

Participants completed 18 sessions over the 6-week training programme. Each session included approximately 30 min of nine-square exercise and 30 min of warm-up and cool-down stretches to improve flexibility and prevent muscle injury. These stretches targeted the upper extremities, trunk and lower extremities (10). We monitored participants for any adverse events during exercise, including swaying, sudden stopping, an inability to perform when returning to exercise after resting, intense pain, muscle soreness, RPE and fear of re-injury.

#### Control Group

All participants continued their normal activities and we did not provide them with a rehabilitation programme.

#### Adherence

We calculated exercise adherence using the percentage of participation in the 18 nine-square exercise sessions.

#### Dynamic Postural Control

We conducted the mSEBT-PM to measure dynamic postural control. The mSEBT-PM is a reliable clinical balance test (ICC = 0.89 [right], 0.97 [left]) (21) and indicates functional performance in individuals with CAI (22). The PM direction is the most representative direction to classify performance between healthy individuals and those with CAI (21, 22). We instructed participants to perform a hands-on-hips, single-leg stance on the CAI limb at the centre of the grid line. Concurrently, the participants reached as far as possible with the untested limb on the PM line while maintaining their postural control. Before testing, they performed six practiced trials with a 10-sec rest between trials. We used the mean of the three-trial reach distance, normalised by leg length (length from the anterior superior iliac spine to the distal tip of the medial malleolus), for data analysis (21, 22).

#### Static Postural Control

The FLT is a reliable test (ICC = 0.97) to measure static postural control in participants with CAI (13, 21). The participants maintained a single-leg stance on the CAI limb with their eyes closed while touching the untested limb at the mid-calf for 30 sec. We counted all lifts of any part of the foot (toes or heel) from the floor. We used the mean of the three trials for data analysis (13, 21).

#### Perceived Ankle Stability

The CAIT is a self-reported ankle stability questionnaire that consists of nine items with scores ranging from 0 to 30. The low score indicates severe ankle instability (15, 23). A cut-off score of ≤ 25 is used to identify CAI (15). The CAIT has an excellent test-retest reliability (ICC [2, 1] = 0.96). The minimal detectable change (MDC) and minimal clinically important difference (MCID) for the CAIT scores were ≥ 3 (23).

#### Self-Reported Ankle and Foot Function

The Foot and Ankle Ability Measure (FAAM) examines ankle and foot functionality with the 21-item activity of daily life (FAAM-ADL) and the eight-item sport (FAAM-Sports) subscales (24). Each item is scored on a 5-point Likert scale from 0 (unable to do) to 4 (no difficulty at all) and with a non-applicable option that is transformed to a percentage from 0 (the greatest disability) to 100 (the least disability). A score below 90% in the FAAM-ADL and below 80% in the FAAM-Sports indicates poor ankle and foot functionality (3, 14). Both the FAAM-ADL (ICC [2, 1] = 0.80) and the FAAM-Sports (ICC [2, 1] = 0.77) have good test-retest reliability (24). The MDC was 3.96% for the FAAM-ADL and 7.90% for the FAAM-Sports (25).

### Statistical Analyses

All statistical analyses were performed with SPSS software version 26.0 for Windows. Some data were missing, as one participant cancelled the last training session and did not complete the post-intervention measurement due to a recurrence of LAS after a contact injury. We included the participant who did not complete all 18 sessions in our intention-to-treat analysis. We used a paired *t*-test to evaluate within-group differences. To evaluate between-group differences, we conducted an analysis of covariance (ANCOVA) to compare the post-intervention scores, using the pre-intervention baseline measures of each outcome variable as covariates. We interpreted Cohen’s *d* and partial η^2^ effect sizes as either large (≥ 0.80), medium (0.50–0.79) or small (0.20–0.49) (26). We set statistical significance at *P* < 0.05.

## Results

Table 1 shows the demographic characteristics of all participants. In the nine-square exercise group, adherence to the protocol was 99.38%, as one participant did not attend the final session because of a sports injury. No participants reported adverse events during the nine-square exercise sessions.

As shown in Table 2, the nine-square exercise group demonstrated significant improvements from baseline to post-intervention in their mSEBT-PM (*P* = 0.004), FLT (*P* = 0.001), CAIT (*P* < 0.001), FAAM-ADL (*P* = 0.038) and FAAM-Sports (*P* = 0.004) scores, with large effect sizes (Cohen’s *d* range: 0.83–2.41). For the control group, the only significant improvement was on FLT scores (*P* = 0.018).

Table 3 presents the ANCOVA results for the adjusted baseline outcomes. These results show that the nine-square exercise group had significantly improved mSEBT-PM (F_1,15_ = 26.19, *P* < 0.001), CAIT (F_1,15_ = 14.69, *P* = 0.002), FAAM-ADL (F_1,15_ = 12.72, *P* = 0.003) and FAAM-Sports (F_1,15_ = 6.71, *P* = 0.020) scores relative to the control group. Between-groups effect sizes were small-to-medium (η_p_^2^ = 0.31–0.64). However, we observed no significant differences in FLT scores between groups (F_1,15_ = 0.001, *P* = 0.972).

## Discussion

Nine-square exercise is a balance and gait training that uses multi-directional stepping. Similarly to high-velocity and low-load training methods, it encourages postural control. Like these methods, multi-directional stepping may improve the activation of the synergist and agonist lower-limb muscles and increase neural function, ultimately helping the sensorimotor system adjust postural control strategies to accomplish various types of steps (10–12). Therefore, we hypothesised that individuals with CAI who participated in a 6-week nine-square exercise programme would experience improved postural control and self-reported ankle stability and ankle and foot functionality. The findings support our hypothesis, as we found that the nine-square exercise group significantly improved dynamic postural control, ankle stability, and ankle and foot functionality relative to the control group. Notably, we also observed that nine-square exercise had no adverse events in individuals with CAI. To the best of our knowledge, the present study is the first demonstrating the beneficial effects of nine-square exercise on postural control and self-reported ankle and foot function and stability in individuals with CAI.

Several interventions have been developed to address postural control impairment in CAI individuals (1, 5–6, 8, 13, 16, 27–29). Previous studies observed the effects of 4-week to 6-week balance and/or functional training programmes with mSEBT-PM scores (5, 8, 13, 27–29). The mSEBT-PM is widely used to assess post-intervention dynamic postural control in individuals with CAI. In our study, the mSEBT-PM scores of the nine-square exercise group improved significantly, with a large effect size. We also noted that the nine-square exercise group had a greater reach distance than the control group, with a medium effect size. Similarly, a study of a 6-week balance training programme on seven different materials—exercise mats, Dynair, Bosu, mini tramps, foam rollers, resistance bands and ankle discs—reported improved reach distances using the mSEBT-PM (*P* < 0.001). They reported a large effect size within (Cohen’s *d* = 1.38) and between groups (Cohen’s *d* = 4.55) for the improvements in post-intervention dynamic postural (8). Similarly, other previous studies applied the mSEBT-PM to identify improvements in an intervention group after 4 weeks to 6 weeks of balance training with a wobble board (13), balancing platform (27), joint position sense blocks (29) and other rehabilitation tools (5, 7, 28) relative to a control group, with small-to-large effect sizes. Focusing on previous studies with a similar sample size to ours, the present finding is similar to the results of a study on two 4-week balance training programmes (*n* = 9 per group) as they reported a significant improved mSEBT-PM with moderate-to-large effect sizes at post-intervention in both programmes. However, there was no difference between groups (29). This finding is also similar to that of Sefton et al. (27), in which a 6-week balance platform training programme (*n* = 12) improved dynamic postural control compared to pre-intervention scores and to the control group (*n* = 9).

Regarding static postural control, when we compared our improved post-intervention and baseline FLT scores, the within-group differences resulted in large effect sizes. Interestingly, while our results indicate the greater improved dynamic postural control in nine-square exercise than the control, we found no difference in static postural control between groups. This result differs to those of a study on 4-week ankle rehabilitation programmes using resistance bands and a biomechanical ankle platform system; the study reported rehabilitation groups improved FLT scores better than the control with small-to-large effect sizes (Hedges’ *g* range: 0.17–1.46) (28). This suggests that combining balance and strength training can improve static postural control more than balance or strength training alone. Given that nine-square exercise is mainly focused on balance and gait training, we propose that coupling nine-square exercise with strength training may enhance static postural control in individuals with CAI. This could be investigated in a future study.

A CAIT score reflects the self-perceived stability of the ankle joint in individuals with CAI. The nine-square exercise group had increased post-intervention CAIT scores, with a large effect size. Moreover, the nine-square exercise group had higher post-intervention CAIT scores than the control group. These findings are similar to those of Cruz-Diaz et al. (8), who report significant improvements in post-intervention CAIT scores, with large effect sizes both within (Cohen’s *d* = 2.32) and between groups (Cohen’s *d* = 2.45). Furthermore, other studies reported that balance training improved self-perceived ankle stability in individuals with CAI (23, 28). We also found that the CAIT scores of participants in the nine-square exercise group surpassed MDC and MCID, indicating that the participants’ clinical CAI symptoms had improved, leading to increased self-perceived ankle stability after the intervention (23, 28).

The FAAM quantifies the activities of daily living (ADLs) and sport-related tasks for the foot and ankle that should be considered during CAI rehabilitation (3, 6). After the intervention, we found significantly improved FAAM-ADL (mean difference = 8.28%) and FAAM-Sports (mean difference = 21.72%) scores for the exercise group, with large effect sizes. Previous studies showed average improvement in FAAM-ADL (mean difference: 2.64%–3.24%) and FAAM-Sports (mean difference: 6.88%–12.24%) scores (6, 28). Although the nine-square exercise group had higher FAAM-ADL (mean difference = 10.95%) and FAAM-Sports (mean difference = 12.46%) scores than the control group, this effect size was small. Our results are similar to those of 4-week and 6-week balance and strength training programmes that led to improved FAAM-ADL and FAAM-Sports scores, with small effect sizes (6, 28). Likewise, in a previous study with a similar sample size to ours, a 4-week progressive hop-to-stabilisation balance group (*n* = 9) improved FAAM-ADL and FAAM-Sports with small-to-moderate effect sizes when compared to a traditional single-limb balance group (*n* = 9) (29). In our study, the mean differences both within and between groups reached the MDC (25). This result confirms that nine-square exercise improved self-reported ankle and foot functionality in individuals with CAI.

Cumulatively, our findings provide evidence that a 6-week nine-square exercise programme can improve dynamic postural control and clinically improve self-rated ankle and foot stability and functionality in collegiate athletes with CAI. Furthermore, the nine-square exercise is a novel exercise based on gait initiation and balance training for individuals with CAI that incurs minimal costs and requires minimal equipment. In our study, adherence to the nine-square exercise was good and no adverse events were reported during exercise. Therefore, this exercise can be used as an alternative treatment programme or for home-based rehabilitation for individuals with CAI.

### Limitations

This study has some limitations. First, we aimed to reduce any risk of bias due to the baseline imbalance between groups that may occur in a randomised controlled trial. Therefore, ANCOVA was used to analyse the data to adjust for the covariates and increase the statistical power of the analysis. Hence, the minimum sample size was calculated using a formula for ANCOVA (18). However, our small sample size could limit the statistical power of our analyses compared to previous studies with larger samples (8, 13). Therefore, further studies with larger samples are needed. Second, all participants were male collegiate athletes with CAI. The impact of nine-square exercise on CAI should be examined in other groups (such as females, professional athletes and adolescents) to generalise the current findings. Third, in the present study, no follow-up was done to monitor the long-term effects of nine-square exercise after the completion of a training programme. Further studies should be conducted with long-term follow-up to confirm that these positive effects persist beyond the training period. Finally, our participants performed the nine-square exercise at a moderate intensity; walking speed was increased based on the individual’s performance. Progressive increases in intensity and walking speed may lead to greater improvements in postural control and other self-reported outcomes. However, more research is needed to determine the optimal intensity of progressive exercise.

## Conclusion

The present results show that a 6-week nine-square exercise programme improved dynamic postural control, self-reported ankle stability, and self-reported ankle and foot function in individuals with CAI. Nine-square exercise is suitable to perform in clinical and field-based settings with no adverse events in individuals with CAI. Therefore, the present results support the use of nine-square exercise as an alternative rehabilitation programme for CAI.

## Figures and Tables

**Table 1:** Demographic characteristics of participants (*n* = 18)

Variables	Mean (SD)		*P-*value

	Nine-square exercise (*n* = 9)	Control (*n* = 9)	
Age (years old)	22.89 (5.60)	23.89 (6.58)	0.96^b^
Weight (kg)	70.06 (6.16)	65.33 (8.29)	0.19^a^
Height (cm)	175.89 (5.78)	170.67 (6.84)	0.10^a^
Number of ankle sprains	5.33 (2.18)	6.44 (6.11)	0.79^b^
Number of feeling instability	4.33 (2.92)	3.78 (2.54)	0.68^b^
Number of episodes of ‘giving way”	3.78 (2.68)	3.22 (2.86)	0.65^b^
Sport types (*n*)			
Team sports (basketball, volleyball, football and rugby)	7	7	
Individual sports (running)	2	2	

Notes:

a Independent *t*-test;

b Mann-Whitney U test

**Table 2:** Within-group difference of outcomes between baseline and post-intervention

Outcome variable	Nine-square exercise (*n* = 9)					Control (*n* = 9)				

	Mean (SD)		Mean difference (95% CI) a	*P*-value^a^	Effect size^b^	Mean (SD)		Mean difference (95% CI) a	*P-*value^a^	Effect size^b^
	
	Baseline	Post-intervention				Baseline	Post-intervention			
mSEBT-PM (%)	84.01 (10.70)	94.42 (7.19)	10.40 (4.34, 16.47)	0.004	1.32	76.94 (7.39)	78.57 (5.65)	1.63 (−2.06, 5.31)	0.34	0.34
Foot lift test (errors)	13.26 (4.15)	8.10 (5.19)	−5.16 (−7.63, −2.68)	0.001	1.60	12.85 (5.43)	7.93 (4.12)	−4.93 (−8.76, −1.09)	0.018	0.99
CAIT (score)	16.00 (3.12)	22.63 (3.97)	6.63 (4.52, 8.74)	< 0.001	2.41	18.33 (2.92)	19.43 (3.60)	1.10 (−0.86, 3.05)	0.23	0.43
FAAM (%)										
ADL subscale	86.64 (10.20)	94.92 (3.47)	8.28 (0.58, 15.98)	0.038	0.83	85.98 (8.03)	83.84 (8.56)	−2.14 (−9.43, 5.16)	0.52	0.23
Sports subscale	65.97 (13.21)	87.69 (7.51)	21.72 (9.05, 34.38)	0.004	1.32	71.53 (5.51)	75.89 (11.24)	4.36 (−1.96, 10.69)	0.15	0.53

Notes: mSEBT-PM = modified star excursion balance test in posteromedial direction; CAIT = Cumberland ankle instability tool; FAAM = Foot and Ankle Ability Measure; ADL = Activities of daily living;

a Paired *t*-test;

b Cohen’s *d* effect size

**Table 3:** The adjusted mean for the outcomes at post-intervention for the nine-square exercise group and the control group

Outcome variable	Nine-square exercise (*n* = 9)			Control (*n* = 9)			Adj mean difference between groups (95% CI)	*F*	*P-*value a	Effect size^b^

	Baseline mean (SD)	Post-intervention		Baseline mean (SD)	Post-intervention					
	
		mean (SD)	Adj mean		mean (SD)	Adj mean				
mSEBT-PM (%)	84.01 (10.70)	94.42 (7.19)	92.67	76.94 (7.39)	78.57 (5.65)	80.32	12.34 (7.20, 17.48)	26.19	<0.001	0.64
Foot lift test (errors)	13.26 (4.15)	8.10 (5.19)	7.98	12.85 (5.43)	7.93 (4.12)	8.05	−0.06 (−3.91, 3.79)	0.001	0.972	0.00
CAIT (score)	16.00 (3.12)	22.63 (3.97)	23.68	18.33 (2.92)	19.43 (3.60)	18.38	5.30 (2.35, 8.25)	14.69	0.002	0.50
FAAM (%)										
ADL subscale	86.64 (10.20)	94.92 (3.47)	94.86	85.98 (8.03)	83.84 (8.56)	83.91	10.95 (4.41, 17.49)	12.72	0.003	0.46
Sports subscale	65.97 (13.21)	87.69 (7.51)	88.02	71.53 (5.51)	75.89 (11.24)	75.56	12.46 (2.21, 22.71)	6.71	0.020	0.31

Notes: mSEBT-PM = modified star excursion balance test in posteromedial direction; CAIT = Cumberland ankle instability tool; FAAM = Foot and Ankle Ability Measure; ADL = Activities of daily living; Adj Mean = mean adjusted for pre-intervention values;

a Analysis of covariance (ANCOVA);

b Partial eta-squared (*η_p_^2^*) effect size
